# Four Different Carotid Atherosclerotic Behaviors Based on Luminal Stenosis and Plaque Characteristics in Symptomatic Patients: An in Vivo Study

**DOI:** 10.3390/diagnostics9040137

**Published:** 2019-10-02

**Authors:** Hiroko Watase, Gador Canton, Jie Sun, Xihai Zhao, Thomas S. Hatsukami, Chun Yuan

**Affiliations:** 1 Department of Surgery, University of Washington, United States 850 Republican Street, Seattle, WA 98109, USA; hiroko7@uw.edu (H.W.); tomhat@uw.edu (T.S.H.); 2 Department of Radiology, University of Washington, United States 850 Republican Street, Seattle, WA 98109, USA; gcanton@uw.edu (G.C.); sunjie@uw.edu (J.S.); 3 Center for Biomedical Imaging Research, Department of Biomedical Engineering, Tsinghua University School of Medicine, China Haidian District, Beijing 100084, China; xihaizhao@tsinghua.edu.cn

**Keywords:** vessel wall magnetic resonance imaging, carotid atherosclerotic plaque, high-risk plaque, carotid stenosis

## Abstract

Correct stratification of ischemic stroke risk allows for the proper treatment of carotid atherosclerotic disease. We seek to differentiate plaque types based on stenosis level and plaque morphology. The Chinese Atherosclerosis Risk Evaluation (CARE–II) study is a cross-sectional, observational, multicenter study to assess carotid atherosclerotic plaques in symptomatic subjects using vessel wall magnetic resonance imaging. Plaque morphology and presence of plaque components were reviewed using multi-contrast magnetic resonance imaging. The carotid arteries were divided into four groups based on stenosis level and plaque components. Out of 1072 ischemic stroke subjects, 452 ipsilateral side carotid arteries were included. Significant stenosis (SS) (≥50% stenosis) with high-risk plaque (HRP) features was present in 37 arteries (8.2%), SS(+)/HRP(−) in 29 arteries (6.4%), SS(−)/HRP(+) in 57 arteries (12.6%), and SS(−)/HRP(−) in 329 arteries (72.8%). The prevalence of SS(−)/HRP(+) arteries in this cohort was substantial and had greater wall thickness than the SS(+)/HRP(−) group. These arteries may be misclassified for carotid revascularization by current guidelines based on the degree of luminal stenosis only. These findings have implications for further studies to assess stroke risk using vessel wall imaging.

## 1. Introduction

Approximately 15–20% of ischemic strokes derive from carotid atherosclerotic plaques [1]. Currently, the most widely used estimator of ischemic stroke risk in both symptomatic and asymptomatic patients is the degree of carotid stenosis [2,3,4,5]. Rupture of atherosclerotic plaque in the carotid artery is believed to be the main source of ischemic embolic cerebrovascular events, including stroke and transient ischemic attacks [6]. This has led to many ground-breaking studies that link plaque rupture to compositional features. The ability of magnetic resonance imaging (MRI) to characterize plaque components is now well established and multiple MRI based prospective studies have identified the plaque features linked to the development of future clinical events. These features have been defined as: plaques with thinned or ruptured fibrous cap, presence of intraplaque hemorrhage (IPH), and presence of a large lipid-rich necrotic core (LRNC) [7,8,9,10,11,12,13,14]. Thus, the presence of these features in MRI may be considered as indicators of high-risk plaque (HRP) for ischemic stroke. Several studies have also indicated that measurement of stenosis alone underestimates plaque burden [15,16]. Further evidence of the discrepancies between stenosis and stroke risk are found in studies examining patients diagnosed with cryptogenic strokes, which found plaques with HRP features in carotid arteries with < 50% stenosis [17,18]. These findings suggest that there may be carotid-related strokes that are missed due to risk assessments based on stenosis levels [17,18,19,20,21]. In the CARE-II (Chinese Atherosclerosis Risk Evaluation) study, 1072 ischemic stroke subjects with evidence of carotid atherosclerotic plaques underwent MRI scans of the carotid arteries [22,23]. The current study examines the relationships between plaque morphology, stenosis levels, and location of plaques in the ipsilateral side of carotid arteries to identify groups whose risk may be miscategorized by current guidelines [2,3,4,5].

## 2. Materials and Methods

### 2.1. Subject Enrollment

This is a cross-sectional study from the CARE–II study data (NCT02017756). The overall design and objectives of CARE–II have been published [22,23]. In brief, CARE–II is an observational, multicenter study to assess carotid atherosclerotic plaque using standardized carotid MRI in a Chinese population. Inclusion criteria were: (1) 18–80 years of age; (2) history of anterior circulation (carotid territory) cerebral hemispheric ischemic symptoms or amaurosis fugax, including ischemic stroke and transient ischemia attack in the previous 14 days; and (3) atherosclerotic plaque in at least one carotid artery, as determined by B-mode ultrasound (intima–media thickness ≥ 1.5 mm). Exclusion criteria were: (1) evidence of cardioembolic stroke; (2) hemorrhagic stroke; (3) history of radiation therapy in the neck; (4) claustrophobia; and (5) contraindication to MR imaging. Clinical characteristics from the time of the hospital visit for the recent ischemic symptoms were acquired from medical records. History of hypertension (defined as diastolic blood pressure ≥ 90 mm Hg or systolic blood pressure ≥ 140 mm Hg), hyperlipidemia (defined as elevated concentrations of any or all of the lipids in the plasma, such as low density lipoprotein >140 mg/dL, total cholesterol >200 mg/dL, or triglycerides >150 mg/dL), diabetes mellitus (fasting blood sugar level ≥ 126 mg/dL, 2-hour oral glucose tolerance test result ≥ 200 mg/dL, or hemoglobin A1c ≥ 6.5%), smoking (current or former), statin use, and coronary heart disease (myocardial infarction or angina) was collected. Institutional review board approvals were obtained for the entire study and from each participating institution, and all study participants provided written informed consent prior to enrollment.

### 2.2. Magnetic Resonance (MR) Imaging

Participating radiologists and MR technologists from each imaging site were trained on carotid MRI acquisition and quality evaluation. A standardized carotid MR imaging protocol was implemented for carotid plaque imaging at all 13 participating centers. All carotid MR imaging was performed on 3.0 T MR scanners with dedicated 8-channel phase–array carotid coils.

### 2.3. Image Analysis

**Figure 1:**
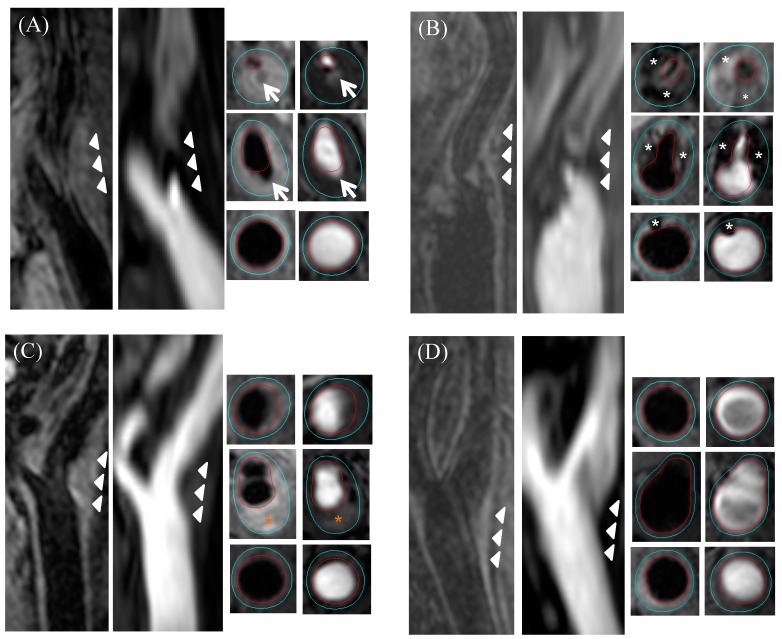
Example magnetic resonance images of the four groups. (**A**) SS(+)/HRP(+): significant stenosis with ulceration (white arrow); (**B**) SS(+)/HRP(−): significant stenosis with calcified plaque (white asterisk); (**C**) SS(−)/HRP(+): no significant stenosis with IPH (orange asterisk); (**D**) SS(−)/HRP (−): no significant stenosis with small plaque. Red outline, lumen; light blue outline, outer vessel wall; white arrowheads, outer vessel wall. (SS = significant stenosis, HRP = high-risk plague, IPH = intraplaque hemorrhage).

Two experienced reviewers who were blinded to the demographic and clinical information analyzed the bilateral carotid artery images with consensus. Image quality was rated per artery on a four-point scale (1 = poor, 4 = excellent). Images with an image quality rating of less than 2 were excluded from this study. Different contrast weighted images were registered during image review using the carotid bifurcation as a reference. Arteries without a carotid bifurcation within the field-of-view were excluded. Lumen and outer wall boundaries were outlined manually on all axial images in 16 mm coverage (8 mm distal and proximal to bifurcation) using CASCADE, a custom-designed image analysis software package (University of Washington, Seattle, WA, USA) [24]. The presence/absence and areas of plaque components, including LRNC, calcification, IPH, and luminal surface disruption (fibrous cap rupture or ulceration) were identified and measured using published criteria [25,26]. HRP was defined as presence of IPH, large LRNC (occupying more than 40% of the wall area), or luminal surface disruption. The minimum lumen area, maximum total vessel area, maximum wall thickness, and eccentricity index at the greatest maximum wall thickness level in the ipsilateral side to ischemic symptoms were analyzed. Eccentricity index was calculated in the axial image per slice: (maximum wall thickness–minimum wall thickness)/maximum wall thickness [27,28]. Three-dimensional time-of-flight MR angiographic images were used to measure carotid stenosis using the NASCET criteria [29]. This analysis was performed independently of carotid plaque morphology and composition measurements. Arteries were classified into four groups (Figure 1): significant (≥ 50%) stenosis (SS) (+)/HRP(+): ≥ 50% stenosis and presence of HRP; SS(+)/HRP(−): ≥ 50% stenosis and absence of HRP; SS(−)/HRP(+): < 50% stenosis and presence of HRP; and SS(−)/HRP(−): < 50% stenosis and absence of HRP.

### 2.4. Statistical Analysis

The summary statistics of the data are presented as mean with standard deviation for continuous variables and count with percentage for binary variables. Clinical and carotid plaque morphology were compared between the HRP (+) and HRP (−) groups using the Mann–Whitney U test (continuous variables) or Fisher’s exact test (binary variables). Univariate and multivariate linear (continuous variables) and logistic (binary variables) regression analyses were performed to assess the differences in carotid plaque morphology between HRP (+) and HRP (−) arteries after accounting for differences in clinical risk factors. Sex is an important factor for atherosclerotic carotid disease [30,31]. Although no significant difference was found, sex was included in the multivariate analysis for the both stenosis groups. In addition, age, hypertension and low density lipoprotein cholesterol (LDL) level were adjusted in the significant stenosis group, and age, smoking, and diabetes mellitus were in the non-significant stenosis group. Although height also showed a significant difference between the two arteries in the significant stenosis group, it is strongly associated with sex. Therefore, height was not included in the multivariate analysis. Throughout the analysis, a two-sided *p*-value (*p*) < 0.05 was considered statistically significant without adjustment for the number of comparisons for this initial hypothesis-generating study. All data analyses were preformed using STATA/SE version 15.1 software (StataCorp; College Station, TX, USA).

## 3. Results

### 3.1. Clinical Characteristics

Of the 1072 recruited subjects, 1047 subjects had bilateral carotid vessel wall MRI with sufficient image quality, of whom 595 subjects were excluded because of missing ischemic symptom side information or time-of-flight image, or insufficient coverage on vessel wall MRI. Among the 452 ipsilateral arteries, 37 (8.2%) arteries were in SS(+)/HRP(+), 29 (6.4%) arteries in SS(+)/HRP(−), 57 (12.6%) arteries in SS(−)/HRP(+), and 329 (72.8%) arteries in SS(−)/HRP(−). Demographic and clinical characteristics of this study population are included in Table 1. Subjects with HRP were older and greater height and LDL level with a higher prevalence of history of hypertension in the SS (+) groups, and older, smoking, and diabetes in the SS(−) groups as compared to those without HRP. Although no significant difference was found, males tended to have HRP compared to females in both significant and non-significant stenosis groups.

### 3.2. Comparison of Plaque Morphology

**Figure 2:**
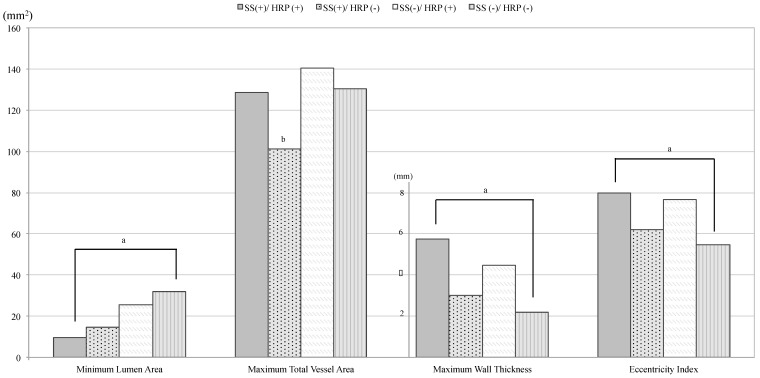
Comparison of plaque morphology between the four groups. ^a^ Values were significant different with each other ^b^ Only ss(+)/HRP(−) group was significant different from the other three groups.

Table 2 and Table 3 summarizes the plaque morphological features between HRP (+) and HRP (−) arteries in the SS (+) and SS (−) groups, respectively. In both SS(+) and SS(−) groups, HRP(+) arteries had a smaller lumen area, greater wall thickness, and eccentricity index compared to the HRP(−) group (all *p* < 0.05) before and after adjusting clinical factors. As shown in Figure 2, these measurements in each group were significantly different. SS(+)/HRP(+) had the smallest lumen, greatest wall thickness, and eccentricity index. Minimum lumen areas in SS(+)/HRP(−) were significantly smaller than in SS(−)/HRP(+) (14.7 ± 9.7 mm^2^ vs. 25.4 ± 11.6 mm^2^, *p* < 0.001), but wall thickness was thinner (3.0 ± 1.4 mm vs. 4.5 ± 1.5 mm, *p* < 0.001). In addition, total vessel areas in SS(+)/HRP(−) were significantly smaller, compared to the other three groups. 

### 3.3. Comparison of Location of Maximum Wall Thickness

When location of plaque was compared between SS(+) and SS(−) arteries and about 60% of SS(+) arteries had plaque in the internal carotid (ICA) segment, whereas about 80% of SS(−) artery had plaque in the common carotid (CCA) segment (*p* < 0.001) (Table 3).

## 4. Discussion

In this study, we sought to determine the differences between the degree of stenosis and presence of HRP in the carotid artery ipsilateral to a recent anterior circulation ischemic cerebrovascular event using MR plaque imaging. The arteries were classified into the four groups based on luminal stenosis, as currently used in clinical practice, and HRP status. Examining the plaque morphology (lumen area, total vessel area, wall thickness, and eccentricity index) within the four groups (Table 2 and Figure 2), an interesting trend emerges. Vessel wall measurements, including wall thickness and eccentricity index, followed a clear descending trend: SS(+)/HRP(+), SS(−)/HRP(+), SS(+)/HRP(−), and SS(−)/HRP(−). However, lumen area did not follow this trend. This difference strongly suggests the need for a new way of assessing the severity of atherosclerosis through not only the stenosis level but also the vessel wall measurements (i.e., wall thickness). This confirms a previous report based on the same data set, where the authors argue that wall thickness measurement is a better predictor of HRP than the degree of stenosis [23].

The SS(+)/HRP(−) arteries represent about 6% of the total cohort. Although current guidelines suggest carotid revascularization in ≥ 50% stenosis of symptomatic patients, vessel wall MRI in the SS(+)/HRP(−) group showed mild wall thickness and smallest total vessel area (Table 2 and Figure 2). Notably, this group showed mostly negative remodeling with significantly small total vessel area. In addition, Hosseini et al. has reported that 179 symptomatic patients with ≥ 50% carotid stenosis and absence of IPH had an estimated annual absolute stroke risk of only 0.6% [7]. Further studies are needed to determine if the arteries in the SS(+)/HRP(−) group (significant luminal stenosis and absence of HRP) are better served with medical management rather than carotid revascularization.

SS(−)/HRP(+) arteries were identified in a relatively large number (13%) of the total cohort. Wall thickness and eccentricity indexes in this group were significantly larger than in the SS(+)/HRP(−) group, whereas lumen areas did not indicate severe stenosis. Thus, increasing wall thickness resulting in outer vessel wall expansion may lead to eccentric plaques. This phenomenon suggests that positive remodeling accounted for the absence of significant luminal stenosis despite increasing wall thickness. Although this group is now categorized as not needing surgical intervention by current clinical guidelines, this group may represent a higher risk group based on vessel wall MRI. Carotid arteries in patients with embolic stroke of undetermined source could plausibly belong to this SS(−)/HRP(+) group [17,18,20]. Further investigation into the risk profiles for this group are needed to develop predictive models that incorporate clinical and hemodynamic data.

Systemic factors including older age, smoking, hypertension, and diabetes were associated with presence of HRP (Table 1), which had been previously reported in symptomatic and asymptomatic patients with mild to severe stenosis [30,32,33,34]. Thus, when HRP was present in the ipsilateral side artery, the contralateral side artery was more likely to have HRP, compared to those without HRP in the ipsilateral side artery (odds ratio 5.4 [95% confidence interval: 2.8–10.3], *p* < 0.001) (not shown in the table). When the location of plaque was compared between SS(+) and SS(−) arteries, SS(+) arteries more commonly had plaque in the ICA segment and more SS(−) arteries had plaque in the CCA segment (*p* < 0.001) (Table 3). This result might indicate that the CCA segment underwent positive remodeling, whereas the ICA segment demonstrated negative remodeling behaviors, which are consistent with previous reports [35,36,37,38]. The location of plaque varied between and within individuals [39] and might be influenced by hemodynamic conditions or carotid bifurcation anatomy, including absolute vessel size, diameter, and area ratios in the CCA, ICA, and external carotid artery [40,41]. Therefore, presence of HRP might be influenced by systematic risk factors, and presence of SS might be influenced by location of plaque and local geometric factors.

This study has several limitations. First, ischemic stroke might not always be secondary to large-artery atherosclerosis [42]. In the future, the ipsilateral carotid arteries in subjects with ischemic stroke due to large artery atherosclerosis only should be analyzed based on this four-group ranking. Second, carotid arteries with 16 mm coverage (8 mm distal and proximal to bifurcation) were included in this sub-analysis study. Plaques outside of this coverage may be missed, but the same range for all carotid arteries was analyzed to compare the vessel morphology. If we change the inclusion criteria to 2 cm coverage, no HRP (+) arteries will be added and the total sample size will be decreased by 14%. We think that 16 mm coverage (8 mm distal and proximal to the bifurcation) is suitable to test the hypothesis in this study, but large coverage 3D imaging techniques that have been recently developed could be more suitable for clinical applications [43,44]. Third, the measurement of luminal stenosis was based on angiographic images derived from a three-dimensional time-of-flight imaging sequence. MR angiography may overestimate carotid artery stenosis compared to digital subtraction angiography, which is the gold standard for measuring carotid artery stenosis. Fourth, the results of this study should be interpreted cautiously because *p*-values were not adjusted for number of comparisons. Therefore, this analysis should be considered hypothesis generating. Lastly, this is a cross-sectional study; thus, it lacks data on the role of HRP features and luminal stenosis in plaque progression and their changes over time. Prospective studies are needed to properly stratify stroke risk using this four-group ranking, especially in SS(+)/HRP(−) and SS(−)/HRP(+) groups.

## 5. Conclusions

By comprehensive evaluation of carotid vessel lumen and wall size and assessing the presence of HRP features in a cohort of subjects with anterior circulation ischemic cerebrovascular symptoms, we found that carotid arteries ≥ 50% or < 50% can be further divided into those with and without HRP. The prevalence of SS(−)/HRP(+) arteries in this cohort was substantial and had greater wall thickness than the SS(+)/HRP(−) group. Presence of HRP was associated with clinical risk factors such as older age, male sex, smoking, and diabetes, whereas stenosis level appears to be influenced by the location of plaque. Further prospective studies are needed to validate these findings and stratify future stroke risk in these four groups.

## Figures and Tables

**Table 1:** Clinical characteristics of 452 subjects.

	SS (+)			SS (−)		
	HRP(+) (37)	HRP (−) (29)	*p*-value	HRP (+) (57)	HRP (−) (329)	*p*-value
Age, y	66.1 ± 8.4	56.4 ± 11.8	**<0.001**	63.7 ± 9.2	61.0 ± 10.3	**0.04**
Sex, male	33 (89.2)	20 (69.0)	0.06	45 (79.0)	219 (66.6)	0.07
BMI, kg/m^2^	25.4 ± 2.6	24.4 ± 2.7	0.19	24.8 ± 3	24.7 ± 3.1	0.64
Height, cm	168.8 ± 4.9	166.1 ± 5.9	**0.03**	167.8 ± 6.4	166.8 ± 7.5	0.41
Smoking	28 (75.7)	17 (58.6)	0.19	38 (66.7)	149 (45.3)	**0.004**
Hypertension	33 (89.2)	19 (65.5)	**0.03**	45 (79.0)	243 (73.9)	0.51
Hyperlipidemia	24 (64.9)	17 (58.6)	0.62	37 (64.9)	188 (57.1)	0.31
LDL, mg/dl	123.0 ± 36.7	106.6 ± 33.5	**0.04**	116.7 ± 38.2	114.2 ± 38.3	0.64
HDL, mg/dl	42.2 ± 8.9	44.2 ± 12.8	0.79	41.2 ± 9.8	44.3 ± 19.5	0.21
TC, mg/dl	183.1 ± 47.4	178.7 ± 41.1	0.54	183.0 ± 42.2	181.2 ± 43.9	0.79
TG, mg/dl	174.8 ± 110.1	144.1 ± 72.3	0.37	169.0 ± 79.0	158.3 ± 90.5	0.14
Diabetes mellitus	13 (35.1)	9 (31.0)	0.80	24 (42.1)	92 (28.0)	**0.04**
Coronary heart disease	10 (27.0)	6 (20.7)	0.59	9 (15.8)	45 (13.7)	0.68
On statin	21 (56.8)	12 (41.4)	0.32	30 (52.6)	138 (42.0)	0.15

Values are mean ± SD or *n* (%). Significant difference (*p* < 0.05) is marked in bold face. SD, standard deviation; BMI, body mass index; LDL, low density lipoprotein cholesterol; HDL, high density lipoprotein cholesterol; TC, total cholesterol; TG, triglyceride; SS, significant stenosis, HRP, high-risk plaque.

**Table 2:** Comparison plaque morphology between high-risk plaque (HRP) (+) and (−) arteries.

			Univariate		Multivariate	
			β or Odds ratio (95%CI)	*p*-Value	β or Odds ratio (95%CI)	*p*-Value
**Significant stenosis group**	**HRP (+)** **(37)**	**HRP (−)** **(29)**				
Minimum lumen area, mm^2^	9.4 ± 6.2	14.7 ± 9.7	−5.2 (−9.2, −1.3)	**0.01**	−5.4 (−10.0, −0.8)	**0.02**
Maximum total vessel area, mm^2^	128.6 ± 39.9	101.3 ± 27.0	27.3 (10.0, 44.5)	**0.002**	26.2 (7.1, 45.3)	**0.008**
Maximum wall thickness, mm	5.8 ± 1.6	3.0 ± 1.4	2.8 (2.0, 3.5)	**<0.001**	2.5 (1.6, 3.4)	**<0.001**
Eccentricity index	0.8 ± 0.1	0.6 ± 0.2	0.2 (0.1, 0.2)	**<0.001**	0.2 (0.1, 0.2)	**<0.001**
**No significant stenosis group**	**HRP (+)** **(57)**	**HRP (−)** **(329)**				
Minimum lumen area, mm^2^	25.4 ± 11.6	31.9 ± 10.7	−6.5 (−9.6, −3.5)	**<0.001**	−6.9 (−10.0, −3.9)	**<0.001**
Maximum total vessel area, mm^2^	140.6 ± 39.0	130.5 ± 36.4	10.2 (−0.2, 20.5)	0.055	6.9 (−3.2, 17.0)	0.18
Maximum wall thickness, mm	4.5 ± 1.5	2.2 ± 1.0	2.3 (2.0, 2.6)	**<0.001**	2.2 (1.9, 2.5)	**<0.001**
Eccentricity index	0.8 ± 0.1	0.5 ± 0.2	0.2 (0.2, 0.3)	**<0.001**	0.2 (0.2, 0.3)	**<0.001**

Values are mean ± SD, *n* (%) or median (IQR). Significant difference (*p* < 0.05) is marked in bold face. HRP, high-risk plaque; LRNC, lipid-rich necrotic core; SD, standard deviation; IQR, interquartile range.

**Table 3:** Comparison location of plaque.

	Significant Stenosis (SS) (+) (66)	SS (−) (386)	*p*-Value
**Internal carotid (ICA) segment**	39 (59.1)	78 (20.2)	**<0.001**
**Common carotid (CCA) segment**	27 (40.9)	308 (79.8)	

Values are *n* (%).
